# THE EFFECT OF MEDICAID MANAGED LONG-TERM SERVICES AND SUPPORT ON PARTICIPANT QUALITY OF LIFE AND UNMET NEED

**DOI:** 10.1093/geroni/igae098.3590

**Published:** 2024-12-31

**Authors:** Howard Degenholtz, Anny Treat, Keri Kastner, John Yauch

**Affiliations:** University of Pittsburgh, Pittsburgh, Pennsylvania, United States; Sebastian Mann Associates, Pittsburgh, Pennsylvania, United States; University of Pittsburgh, Pittsburgh, Pennsylvania, United States; University of Pittsburgh, Pittsburgh, Pennsylvania, United States

## Abstract

In 2018 the Commonwealth of Pennsylvania began a three year transition of Medicaid Aging and Disabled Waiver programs to Managed Long-Term Services and Supports (MLTSS). The new program, Community HealthChoices, serves adults with disabilities who receive home and community based services (HCBS), live in a nursing facility, and people dually eligible for both Medicaid and Medicare who do not use long-term services and supports (“duals”). Three managed care organizations operate the program through state-wide contracts. The program was implemented statewide over a three year period in three regional phases, creating a unique natural experiment. In other analyses, we have demonstrated significant changes to care management and slowing in the rate of growth of HCBS spending. This is the first study of HCBS participants’ subjective experience. A total of 14,706 prospective telephone interviews were conducted with random samples of participants before the transition to CHC and 18 and 30 months post-transition. We found that psychological well-being was unchanged, but depressive symptoms decreased over time. There was no change in the consequences of unmet need (e.g., having a hot meal or a bath). In general, when compared duals, HCBS users report lower access to primary care and lower self-reported health status. After the implementation, duals users reported higher health status and HCBS users were more likely to report having a medical care coordinator. The transition to MLTSS brought substantial changes to the way Medicaid LTSS is financed and delivered in Pennsylvania. However, the experience of the average participant has been relatively stable.

